# 3,5,4′-tri-O-acetylresveratrol Ameliorates Seawater Exposure-Induced Lung Injury by Upregulating Connexin 43 Expression in Lung

**DOI:** 10.1155/2013/182132

**Published:** 2013-03-12

**Authors:** Lijie Ma, Yanyan Li, Yilin Zhao, Qingwei Wang, Yandong Nan, Deguang Mu, Wangping Li, Ruilin Sun, Faguang Jin, Xueying Liu

**Affiliations:** ^1^Department of Respiration, Tangdu Hospital, Fourth Military Medical University, Xi'an 710038, China; ^2^Department of Pharmacy, Tangdu hospital, Fourth Military Medical University, Xi'an 710038, China; ^3^Department of Medicinal Chemistry, School of Pharmacy, Fourth Military Medical University, Xi'an 710032, China

## Abstract

The aim of the present study was to examine the effects of 3,5,4′-tri-O-acetylresveratrol on connexin 43 (Cx43) in acute lung injury (ALI) in rats induced by tracheal instillation of artificial seawater. Different doses (50, 150, and 450 mg/kg) of 3,5,4′-tri-O-acetylresveratrol were administered orally for 7 days before modeling. Four hours after seawater inhalation, histological changes, contents of TNF-**α**, IL-1**β** and IL-10, and the expression of Cx43 in lungs were detected. Besides, the gap junction communication in A549 cells and human umbilical vein endothelial cells (HUVECs) challenged by seawater was also evaluated. Histological changes, increased contents of inflammatory factors, upregulation in gene level, and deregulation in protein level of Cx43 in lungs stimulated by seawater were observed. On the other hand, pretreatment with 3,5,4′-tri-O-acetylresveratrol significantly inhibited infiltration of inflammation, development of pulmonary edema, and contents of inflammatory mediators in lungs. Above all, 3,5,4′-tri-O-acetylresveratrol upregulated the expression of Cx43 in both gene and protein levels, and its intermediate metabolite, resveratrol, also enhanced the gap junction communication in the two cell lines. The results of the present study suggested that administration of 3,5,4′-tri-O-acetylresveratrol may be beneficial for treatment of inflammatorycellsin lung.

## 1. Introduction

Drowning is the second accidental death causes in the world [1]. It is estimated that more than 500,000 people died from drowning each year. Basically, there are two different outcomes of drowning, death on the spot of drowning, and survival from the initial apnoea. However, with the lower respiratory tract challenged by water, the survivor may suffer acute lung injury (ALI), which is characterized by developing pulmonary inflammation and edema [2]. It was reported that inflammation factor secretion, pulmonary edema, and inflammatory spreading to entire lung or even both lungs were closely related to the alteration of communication between cells [3].

Gap junction channels (GJCs), connecting the cytoplasm between adjacent cells, are cell membrane channels, which provide a pathway for rapid exchange of ions, metabolites, and small intracellular signal molecules, such as Ca^2+^, cyclic AMP, and so on. The critical contribution of GJCs to disease etiology has been intensively researched in recent years [4], and connexin 43 (Cx43), as the main mode of connection between alveolar epithelial cells, participates in a variety of acute/chronic lung disease occurrence and development [5]. Evidence proven that Cx43 may regulate Ca^2+^ signal path way, and this would play a pivotal role in acute lung injury [6].

**Figure 1:**
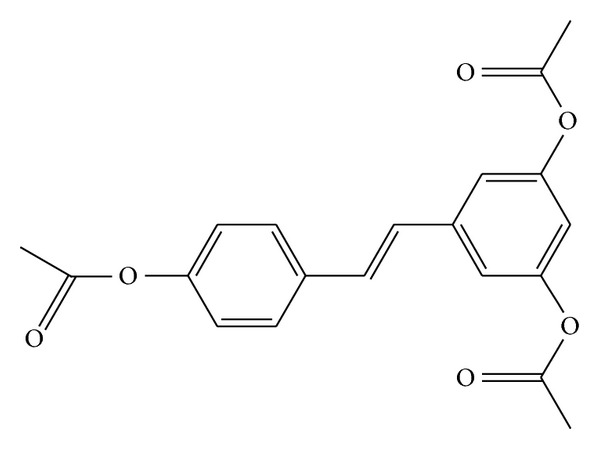
The structure of 3,5,4′-tri-O-acetylresveratrol.

3,5,4′-tri-O-acetylresveratrol (Figure 1), with three hydroxyls replaced by acetyls, is an analog of resveratrol. Several studies demonstrated that it exerted anti-*γ*-irradiation effects by inhibiting the expression of reactive oxygen species (ROS) [7] and acted as inhibitors of PAF-induced washed rabbit platelet aggregation [8]. Besides, our previous results showed that 3,5,4′-tri-O-acetylresveratrol might trigger the accumulation and concentration of resveratrol in lungs [9].

Based on the previous evidence, we put forward and proved in the present research the hypothesis that Cx43 participated in the inflammation induced by seawater instillation via affecting intracellular communication. While 3,5,4′-tri-O-acetylresveratrol could protect lungs by enhancing the expression of Cx43, suppressing inflammatory reaction and reconstructing intercellular communication.

## 2. Materials

### 2.1. Animal Preparation

Male Sprague-Dawley rats, weighing 180–220 g each, were obtained from the Animal Center (Fourth Military Medical University, Xi'an, China). The rats were housed in air-filtered, temperature-controlled units with 12-hour light-dark cycles and had free access to food and water. All experiments were approved by the Animal Care and Use Committee of the Fourth Military Medical University and were in accordance with the Declaration of the National Institutes of Health Guide for Care and Use of Laboratory Animals (Publication No. 85-23, revised 1985).

### 2.2. Reagents

Seawater (osmolality 1300 mmol/L, pH 8.2, SW 1.05, NaCl 6.518 g/L, MgSO_4_ 3.305 g/L, MgCl_2_ 2.447 g/L, CaCl_2_ 1.141 g/L, KCl 0.725 g/L, NaHCO_3_ 0.202 g/L, NaBr 0.083 g/L) was prepared according to the major composition of the East China Sea provided by Chinese Ocean Bureau. 3,5,4′-tri-O-acetylresveratrol was obtained from the Pharmacy Department of Medicinal Chemistry with HPLC purity >99%. Resveratrol was purchased from Xi'an Grass Plant Technology Corporation (Xi'an, China), purity > 98%. Lucifer Yellow CH dilithium salt was purchased from Sigma Chemical Company (St. Louis, MO, USA). Enzyme-linked immunosorbent assay (ELISA) kits of TNF-*α* and IL-1*β* were purchased from R&D Corporation (R&D Systems Inc., Minneapolis, MN, USA). ELISA kit of IL-10 was purchased from SenXiong Science and Technology Industrial Corporation (Shanghai, China). Anti-connexins 43 and anti-*β*-actin monoclonal antibodies were obtained from Anbo Biotechnology Company (Changzhou, China). Real Time PCR related reagents were provided by Takara Biotechnology (Dalian) Co., Ltd. The purity of all chemical reagents was at least at analytical grade.

### 2.3. Modeling and Grouping

SD rats were randomly assigned into 5 groups (*N* = 8).

- Control group: rats without any intervention.
- Seawater drowning group: the rats were anesthetized with pentobarbital sodium (100 mg/kg of body wt, administered i.p.). A heparin-filled blunt-ended polyethylene catheter was inserted into the left carotid artery to monitor the mean arterial pressure and obtain blood samples. After exposure of the trachea, a 20 min stable baseline period was followed, then a syringe (1 mL) was inserted into the trachea and seawater (4 mL/kg) was instilled at a steady speed within 4 min into both lungs. All rats were sacrificed at 4 h after seawater instillation.
- 3,5,4′-tri-O-acetylresveratrol (50 mg/kg) + Seawater drowning group: 3,5,4′-tri-O-acetylresveratrol was administered daily orally for 7 days before modeling.
- 3,5,4′-tri-O-acetylresveratrol (150 mg/kg) + Seawater drowning group: 3,5,4′-tri-O-acetylresveratrol was administered daily orally for 7 days before modeling.
- 3,5,4′-tri-O-acetylresveratrol (450 mg/kg) + Seawater drowning group: 3,5,4′-tri-O-acetylresveratrol was administered daily orally for 7 days before modeling.

The doses of 3,5,4′-tri-O-acetylresveratrol (50, 150, and 450 mg/kg) used here were based on previous dose-response and time-course studies carried out in our laboratory. All rats were anesthetized and exsanguinated through aortic transection 4 hours after modeling. The lungs were moved out rapidly from thoraxes and processed in the manners described below.

### 2.4. Histology

At the end of the experiments, lung tissues of the same lobe from every rat were fixed with 10% formalin for 24 h, and then embedded in paraffin. After deparaffinization and dehydration, the lungs were cut into 5 *μ*m-thick sections with a microtome and stained with haematoxylin and eosin.

### 2.5. Lung Wet-to-Dry Weight Ratio

Lung wet-to-dry ratio (W/D) was used to quantify the magnitude of pulmonary edema. The lung tissues, obtained 4 h after modeling, were weighed immediately, and then dried to constant weight at 70°C for 72 h and weighed again. The wet-to-dry ratio was calculated through dividing the wet weight by the dry weight.

### 2.6. Measurement of Cytokines

Levels of TNF-*α*, IL-1*β*, and IL-10 in the lung tissues were determined by using commercially available ELISA kits. Briefly, lung tissues were homogenized in cool phosphate-buffered saline (lung tissue to PBS 1 : 5). The assay was carried out according to the manufacturer's instructions.

### 2.7. Western Blot Analysis for Cx43

The lungs were perfused with pH 7.4 PBS to remove the blood cells from the pulmonary circulation, and then, the tissue samples from each group were collected and the total proteins were extracted. Protein concentrations were determined by BCA protein assay kit. The protein samples were boiled, separated on a 12% SDS-polyacrylamide gel, electrotransferred to nitrocellulose membranes, blocked with 5% nonfat dry milk in Tris-buffered saline with Tween 20, and incubated overnight at 4°C with monoclonal antibodies against Cx43 (1 : 200) and *β*-actin (1 : 5000). After repeated washing, the secondary antibody (anti-rabbit IgG peroxidase conjugated, 1 : 10000) was incubated, and bands were visualized by using the enhanced chemiluminescence (ECL) system (Amersham Pharmacia Biotech, Arlington Heights, IL, USA). The results were expressed as the ratio to *β*-actin level in the same protein samples.

### 2.8. Determination of Cx43 mRNAs in Lung Tissues

The total RNA of the lung samples was extracted with TRIZOL reagent (Takara). RNA concentration was tested by spectrometric analysis. Cx43 and *β*-actin were examined by Real Time PCR following the manufacturer's instructions (Takara Perfect Real Time). The mRNA of Cx43 gene was normalized to the level of *β*-actin. Genes and primers are listed as follows: Cx43, (forward) 5′-GGAAATCGAACGGCTGGGCGT-3′, (reverse) 5′-TCGCGTGAAGGGAAGAAGCGAT-3′; *β*-actin (forward) 5′-GCACTGTGTTGGCATAGAGGTC-3′, (reverse) 5′-ACGGTCAGGTCATCACTATCGG-3′. Amplification and detection were carried out by using Bio-Rad My iQ detection system (Edinburgh Biological Science and Technology Development co., LTD, Shanghai, China).

### 2.9. Cell Culture and Treatment

The human lung epithelial cell line, A549 (obtained from ATCC, Rockville, MD, USA), was maintained in 1640 medium supplemented with 10% fetal calf serum. The human umbilical vein endothelial cell (HUVEC) line was maintained in ham's F12medium supplemented with 10% fetal calf serum. Both of the two cell lines were treated with 100 U/mL of penicillin and 100 *μ*g/mL of streptomycin at 37°C in a humidified atmosphere containing 5% CO_2_ and 95% air. After incubated in the presence or absence of resveratrol (200 *μ*mol/L), seawater (0.25 mL per 1 mL total volume) was added to A549 and HUVEC cells and the cells were stimulated for the 4 h.

### 2.10. Scrape-Loading and Dye Transfer Technique

A dye transfer assay was used to assess gap junction communication. A549 cells were rinsed with 2 mL PBS after medium was removed from the plates. 2 milliliters of 0.075% Lucifer yellow CH(LY), a fluorescent membrane-impermeable cell marker dye dissolved in PBS, were added to the cells, and two scrape lines (parallel, equidistant scrapes per well) were made by gently passing a diamond-tipped pen (tip diameter, 0.25 mm) across the cultures. The plates were placed for 5 minutes at 37°C in a humidified 5% CO_2_ incubator. The dye solution was then discarded, and the dishes were rinsed twice with PBS to remove background fluorescence. After that, cells were examined with an inverted confocal microscope (FV1000 IX81, Olympus) at emission/excitation wavelengths of 528/425 nm. The images were quantified by counting the number of donor and recipient cells and calculating a cell coupling index with the ratio of recipient to donor cells. The value of dye transfer, defined as the number of secondary recipient cells visualized by Lucifer yellow CH, was recorded for only one side of the scrape (Figure 8).

### 2.11. Statistical Analysis

Statistical analysis was performed with SPSS 13.0 for Windows. Numeric variables are expressed as means ± SD. Statistically significant differences between experimental conditions were performed by one-way analysis of variance (ANOVA) followed by Dunnett's test. A *P* value < 0.05 was considered statistically significant.

## 3. Results

### 3.1. Effects of 3,5,4′-tri-O-acetylresveratrol on Histopathological Changes

**Figure 2:**
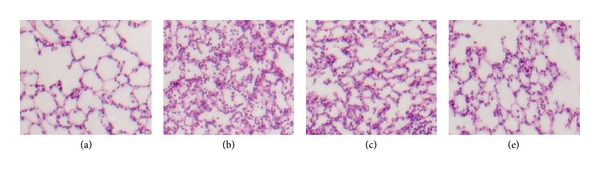
Microscopic findings of lung tissues stained with hematoxylin-eosin (×200). (a) control group; (b) seawater drowning group: edema, hemorrhage, thickened alveolar septum, and infiltration of inflammatory cells were observed in lung samples; (c) 3,5,4′-tri-O-acetylresveratrol (50 mg/kg) group; (e) 3,5,4′-tri-O-acetylresveratrol (450 mg/kg) group. Lung injuries were significantly alleviated by 3,5,4′-tri-O-acetylresveratrol, especially high dose of 3,5,4′-tri-O-acetylresveratrol.

The results showed that 4 hours after seawater inhalation induced pulmonary edema, alveolar damage, and infiltration of inflammatory cells in the lung tissues and alveoli (Figure 2(b)), but pretreatment with different doses of 3,5,4′-tri-O-acetylresveratrol could significantly improve the lung injury (Figures 2(c) and 2(d)).

### 3.2. Effects of 3,5,4′-tri-O-acetylresveratrol on the Lung Edema

**Figure 3:**
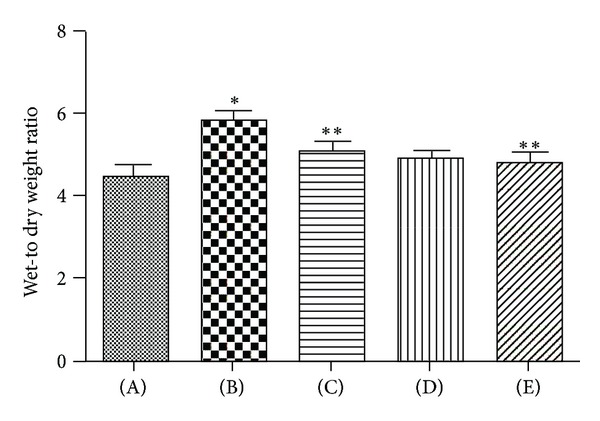
Effects of 3,5,4′-tri-O-acetylresveratrol on lung edema of rats following seawater aspiration. Data are mean ± SD, *n* = 8. (A) control group; (B) seawater drowning group; (C), (D), and (E) different doses of 3,5,4′-tri-O-acetylresveratrol groups. *P < 0.01 versus control group; *P < 0.05 versus **P.

To observe the lung edema, we observed the lung wet/dry weight ratios (Figure 3). The wet/dry ratios significantly increased in Seawater drowning group compared with the control (*n* = 8, *P* < 0.05). However, administration with 3,5,4′-tri-O-acetylresveratrol markedly reduced the lung edema.

### 3.3. Effects of 3,5,4′-tri-O-acetylresveratrol on TNF-*α*, IL-1*β*, and IL-10 Levels

**Figure 4:**
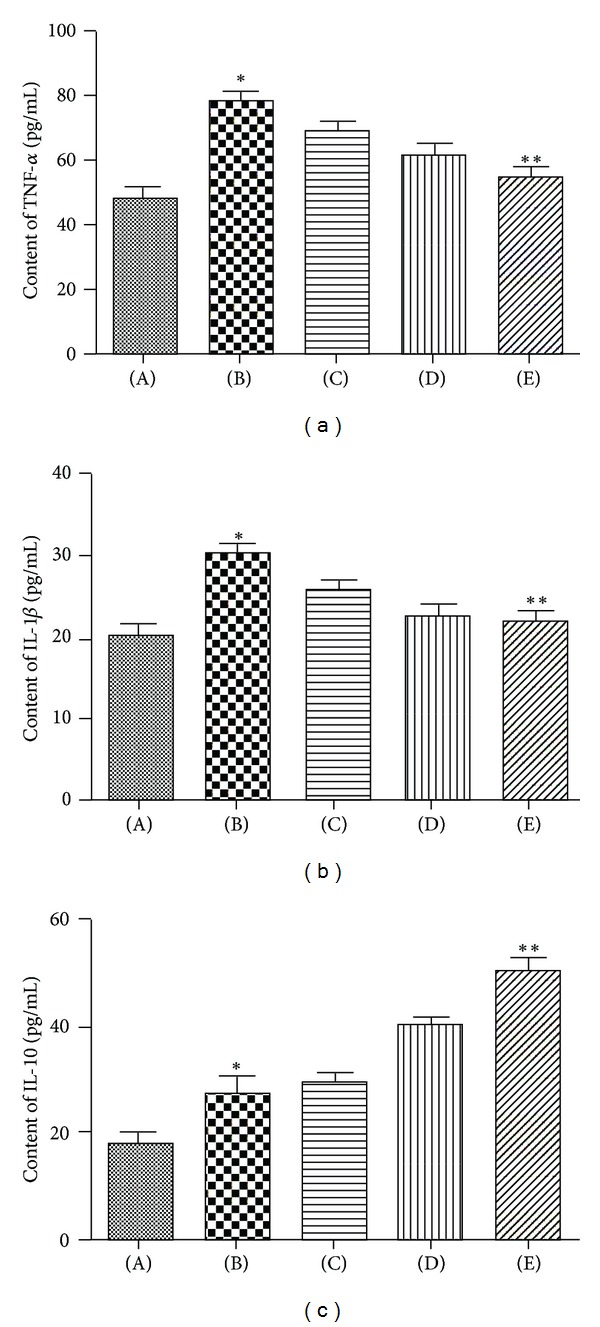
Effects of 3,5,4′-tri-O-acetylresveratrol on TNF-*α* (a), IL-1*β* (b), and IL-10 (c) of lung tissue. (A) control group; (B) seawater drowning group; (C), (D), and (E) different doses of 3,5,4′-tri-O-acetylresveratrol groups. The values of TNF-*α*, IL-1*β*, and IL-10 were increased by seawater instillation. 3,5,4′-tri-O-acetylresveratrol pretreatment downregulated the level of TNF-*α* and IL-1*β* and upregulated the level of IL-10. *P < 0.05 versus control group; *P < 0.01 versus **P.

We also examine the effects of 3,5,4′-tri-O-acetylresveratrol on the levels of TNF-*α*, IL-1*β*, and IL-10 in the lung tissues. As shown in Figure 4, four hours after seawater instillation, TNF-*α* and IL-1*β* contents significantly increased. Pretreatment with 3,5,4′-tri-O-acetylresveratrol markedly inhibited the expression of these inflammatory mediators. Meanwhile, the release of IL-10 was elevated by 3,5,4′-tri-O-acetylresveratrol.

### 3.4. Effects of 3,5,4′-tri-O-acetylresveratrol on Cx43 Expression

**Figure 5:**
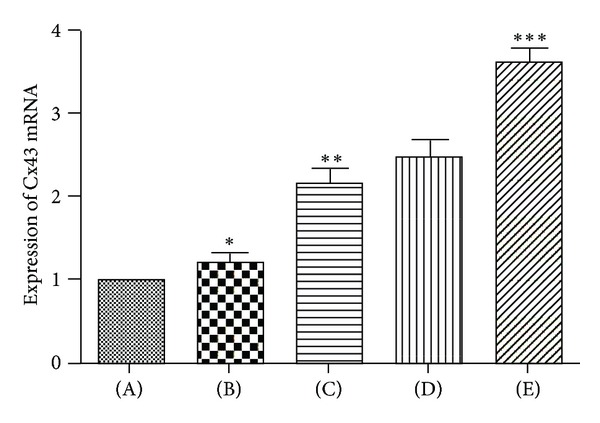
Effects of 3,5,4′-tri-O-acetylresveratrol on Cx43 mRNA expression. The expression level of Cx43 mRNA was taken as “1” in normal lung, and its expression in other groups was compared with the normal expression. (A) control group; (B) seawater drowning group; (C), (D), and (E) different doses of 3,5,4′-tri-O-acetylresveratrol groups. The expression of Cx43 mRNA was increased by seawater, 3,5,4′-tri-O-acetylresveratrol pretreatment upregulated Cx43 mRNA expression. *P < 0.05 versus control group; *P < 0.01 versus **P; **P < 0.01 versus ***P.

Cx43 mRNA expression was examined using Real Time PCR on the lung samples 4 h after seawater exposure (Figure 5). The results showed that seawater aspiration increased the Cx43 mRNA level (*P* < 0.05), while pretreatment with different doses of 3,5,4′-tri-O-acetylresveratrol obviously upregulated the level of Cx43 mRNA (*P* < 0.05).

**Figure 6:**
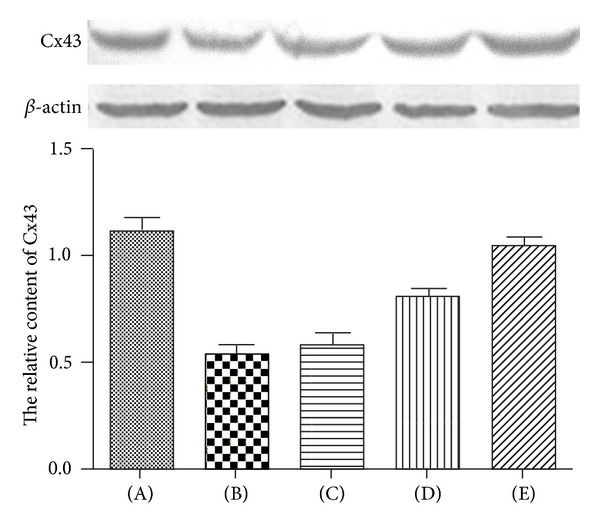
Effects of 3,5,4′-tri-O-acetylresveratrol on Cx43 in seawater stimulated lungs. (A) Control group; (B) seawater drowning group; (C), (D), and (E) different doses of 3,5,4′-tri-O-acetylresveratrol groups. After protein quantitation, western blot was performed to investigate the Cx43 content at 4 h after seawater administration. Ratios of Cx43 protein versus *β*-actin in three independent experiments were obtained by density scanning using an image analysis system. *P < 0.01 versus control group; *P < 0.01 versus **P; **P < 0.01 versus ***P.

We then performed western blot to study the changes of Cx43 in protein levels (Figure 6). When challenged with seawater, the reactive pattern of Cx43 protein was opposite to that of its mRNA. Namely, the protein levels of Cx43 in lungs decreased 4 h after seawater exposure (*P* < 0.05).

### 3.5. Effects of Resveratrol on Dye Transfer in A549 and HUVEC

**Figure 7:**
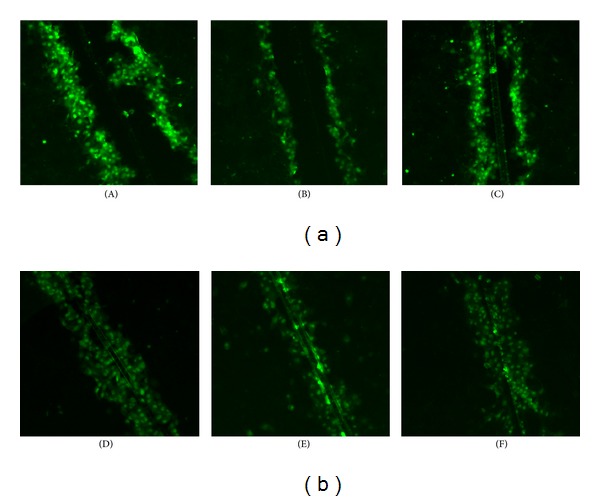
Dye transfer in A549 cells (a) and HUVEC (b) on slides. The panels show a region of A549 cells and HUVEC scrape-loaded with dye ((A) and (D) control; (B) and (E) seawater; (C) and (F) resveratrol). Green indicates Lucifer yellow, including cell initially loaded with dye and recipient cells linked to the donors by gap junctions.

**Figure 8:**
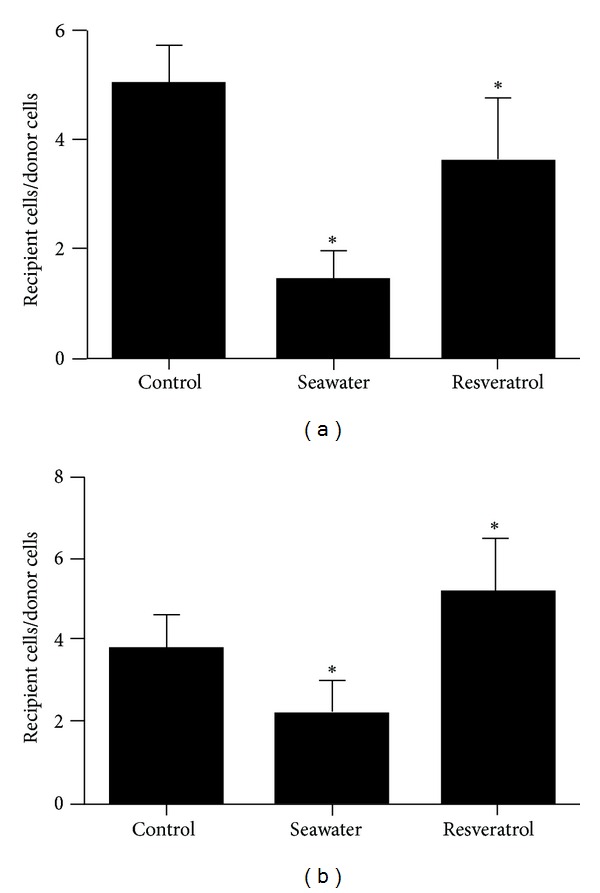
Quantification of dye transfer in G control, G seawater, and G resveratrol (*P < 0.01), (a) A549 cells; (b) HUVEC.

Similar cell numbers were initially loaded with dye in all treatment groups (Figures 7(a) and 7(b)). However, compared with ratios of the control group, there were fewer labeled neighboring cells of seawater groups, and the treatment with resveratrol significantly increased the number of labeled neighboring cells. 

## 4. Discussion

In the present study, we demonstrated that (i) intratracheal instillation of seawater (4 mL/kg) induced obvious histological changes, pulmonary edema, and inflammation in a dosage dependence manner; (ii) seawater stimulation could obviously suppress the cellular communication between cultured cells; (iii) pretreatment with 3,5,4′-tri-O-acetylresveratrol could effectively alleviate the seawater-induced lung injuries; (iv) resveratrol, intermediate metabolite of 3,5,4′-tri-O-acetylresveratrol, could rebuild or strengthen the seawater impaired communication between cells.

Although several promising pharmacological therapies have been studied for patients with ALI and ARDS, none of these pharmacological treatments obviously reduced mortality [10]. Resveratrol (3,5,4′-tri-O-acetylresveratrol) is a polyphenolic compound which is a phytoalexin synthesized by a wide variety of plant species. It has lots of pharmacological properties, such as antioxidation, anti-inflammatory, cardioprotection, cell cycle inhibition, and neuroprotection [11]. However, resveratrol has some shortages of its own. For example, it is not stable with short half-life and it has a low bioavailability [12]. While resveratrol's analog, 3,5,4′-tri-O-acetylresveratrol may overcome some of those disadvantages to some extent. Evidence showed that this analog was effective in inhibiting the expression of reactive oxygen species (ROS) [7] and PAF-induced washed rabbit platelet aggregation [8]. More importantly, it may lead to accumulation and concentration of resveratrol in lungs.

ALI/ARDS can be divided into two categories based on origin: direct (or pulmonary) ALI and indirect (extra-pulmonary) ALI [13]. Seawater drowning-induced acute lung injury (SWD-ALI) belongs to direct ALI. Clinical studies indicated that white blood cell and neutrophils apparently increased in most acute lung injury (ALI)/acute respiratory distress syndrome (ARDS) patients' plasma with bilateral diffuse or localized alveolar infiltrates on chest X-ray [14]. Our previous results also showed that infiltration of inflammatory cells [15] and permeability of alveolar wall to Evans blue [16] apparently increased in SWD-ALI rat model, which to some extent explained the possible mechanism of seawater drowning-induced acute lung injury.

Inflammation response is a series of complex pathological process, including release of cytokines, growth factors and chemokine, and migration of neutrophils, monocytes and lymphocytes to tissue spaces. All the above pathological processes need the coordination of communication between cells. Evidence showed that communication between flanking cells stimulated by Cx43 provided the foundation for the onset and development of inflammation in lung tissues [17]. We found, in the present study, that seawater exposure resulted in upregulation of Cx43 in gene level, deregulation of Cx43 protein, and secretion of inflammatory factors, such as TNF-*α* and IL-1*β*, while pretreatment with 3,5,4′-tri-O-acetylresveratrol reduced the contents of inflammation factor. Besides, IL-10, as a famous inhibitory inflammation factor, was increased by 3,5,4′-tri-O-acetylresveratrol. More importantly, 3,5,4′-tri-O-acetylresveratrol markedly upregulated the expression of Cx43 in gene and protein levels, and its intermediate metabolite contributed to the reconstruction of cellular communication in A549 and HUVEC.

Pulmonary edema is another critical issue of acute lung injury, which results from breathing membrane barrier (BBB) dysfunction, and capillary permeability increase [18, 19]. Accumulation of liquid rich in protein in alveolar space may increase the mortality of ALI/ARDS. It was demonstrated, in a gunshot lung injury rabbit model, that cellular communication played an important role in capillary permeability increase and leakage of liquid into alveolar space [20]. In addition, it was found that inhibition of Cx43 mRNA and protein expression led to the increase of single cell permeability [21]. We found, in the present study, that seawater inhalation led to lung edema and seawater stimulation also suppressed cellular communication of HUVEC. However, 3,5,4′-tri-O-acetylresveratrol could alleviate the lung edema in rats suffering seawater instillation, and its metabolic intermediates, resveratrol, recovered the cellular communication restrained by seawater exposure.

Connexin 43 (Cx43), which plays a key role in regulating of inflammation and microvascular permeability, increased in gene level while was inhibited in protein level upon seawater stimulation. This may mean that seawater stimulation would enhance the transcription but inhibit the translation of Cx43. We observed the facilitating effect of 3,5,4′-tri-O-acetylresveratrol on Cx43 in SWD-ALI and two kinds of cells stimulated by seawater. Our results suggested that 3,5,4′-tri-O-acetylresveratrol alleviated seawater instillation induced inflammation and pulmonary edema probably via activation of Cx43.
